# College and Hospital Notes

**Published:** 1911-02

**Authors:** 


### COLLEGE AND HOSPITAL NOTES

The Buffalo General Hospital in its obstetric wards has a number of free beds for occupancy by maternity patients. We are glad to announce this fact, and to say that needy patients at or near term may be sent to the hospital, where they will receive intelligent and conscientious attention; in fact, each and every such patient will be attended by a competent and specially qualified physician. There are many women near term who, not able to receive proper care at their homes, should avail themselves of this great opportunity.

